# White Nail as a Static Physical Finding: Revitalization of Physical Examination

**DOI:** 10.3390/clinpract11020036

**Published:** 2021-05-01

**Authors:** Ryuichi Ohta, Chiaki Sano

**Affiliations:** 1 Community Care, Unnan City Hospital, 699-1221 96-1 Iida, Daito-cho, Unnan 699-1221, Shimane Prefecture, Japan; 2 Department of Community Medicine Management, Faculty of Medicine, Shimane University, 89-1 Enya cho, Izumo 693-8501, Shimane Prefecture, Japan; sanochi@med.shimane-u.ac.jp

**Keywords:** Lindsay’s nail, nail findings, nutritional assessment, physical examination, Terry’s nail, white nail

## Abstract

Physical examinations are critical for diagnosis and should be differentiated into static and dynamic categories. One of the static findings is white nail, such as Terry’s and Lindsay’s nails. Here, we report the cases of two older patients with acute diseases who had nail changes that aided evaluation of their clinical course. Two elderly women who presented with acute conditions were initially thought to have normal serum albumin levels. They were found to have white nail with differences in nail involvement of the first finger, which subsequently revealed their hypoalbuminemia. The clinical courses were different following the distribution of nail whitening. Our findings show that examination of a white nail could indicate the previous clinical status more clearly than laboratory data. It can be useful for evaluating preclinical conditions in patients with acute diseases. Further evaluation is needed to establish the relationship between clinical outcomes and the presence of white nail in acute conditions among older patients.

## 1. Introduction

Physical examinations are critical not only for diagnosis but also for building a rapport with patients [1,2]. However, most current medical examinations depend on laboratory and imaging tests [3]. Furthermore, physicians and medical trainees conduct fewer physical examinations and interact less with their patients [4,5]. This could result in ineffective therapeutic relationships. Artificial intelligence is likely to replace laboratory and imaging tests in the near future [6,7,8,9]. Therefore, general physicians and trainees should revitalize the importance of physical examinations by categorizing their contents more effectively.

Physical examinations used in clinical situations should be differentiated into static and dynamic categories. This will facilitate medical trainees’ comprehension of their effectiveness. Static and dynamic examinations contribute to specific diagnoses. While history-taking has high sensitivity, physical examination has high specificity [10]. Though they may provide similar information, the former depends on the history-taker’s skills and the use of the information obtained [11]. History-taking indicates the symptoms based on real-time data. However, physical findings change with time [12]. Dynamic physical findings include specific changes in acute diseases, such as the crackles of pneumonia and heart failure, and pitting edema in diseases with volume retention [12]. These directly contribute to disease diagnosis. Conversely, static physical findings can be found in “cumulative” parts of human bodies, such as the nails, eyes, skin, and hair. The typical static nail findings are Terry’s and Lindsay’s nails, which are indicative of hypoalbuminemia, cirrhosis, heart failure, or renal dysfunction [13]. These changes can identify previous clinical conditions which affect the clinical course and those that laboratory data may not detect. The two kinds of nails are differentiated on the basis of the proportion of the white proximal part and distal reddish-brown band. In Lindsay’s nail, the distal reddish-brown band occupies 20–60% of the nail bed and does not fade with pressure, whereas, in Terry’s nail, the distal reddish-brown band is narrow and the proximal white part occupies approximately 80% of the nail bed [14]. Static physical findings can support clinical management. However, there is a lack of evidence supporting the usefulness of static physical findings in older people. In addition, clinical differentiation of Lindsay’s and Terry’s nails in hands is difficult [13,14]. Terry’s and Lindsay’s nails can be defined comprehensively as white nails; however, such changes may not be clearly evident and are often overlooked in older patients. This report describes the cases of two older patients with nail changes and demonstrates the importance of examining white nails.

## 2. Case Presentation

### 2.1. White Nails in All Nails of the Hands

**Figure 1:**
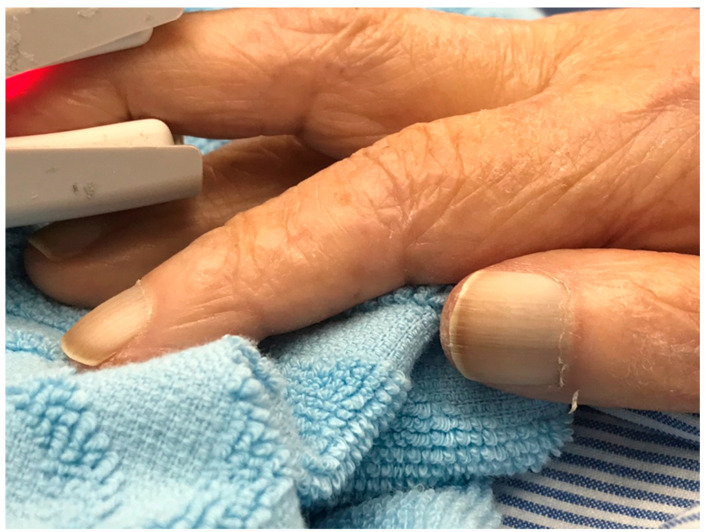
The patient’s white fingernail bed of the right hand.

An 83-year-old woman came to our hospital with chief complaints of mild fever and appetite loss for two weeks. Her past medical history included hypertension and Alzheimer’s disease, without liver disease, renal disease, or heart failure. Her previous activities of daily living (ADL) were not poor, as she could lead her daily life by herself. Her vital signs were normal except for raised temperature (37.8 °C), and she had right costovertebral angle tenderness and pyuria. On admission, her laboratory data showed that the serum albumin concentration was 3.1 g/dL, and the estimated glomerular filtration rate was 53 mL/min/1.73 m^2^. She was diagnosed with a urinary tract infection, treated with cefmetazole 4 g/day for two weeks, and was cured with a full recovery of appetite. All of the fingernail beds of her hands were white (90%) with brown distal portions (10%); this led us to diagnose Lindsay’s nail (Figure 1). Her level of vitamin B1 was 12 μg/dL, and thyroid stimulating hormone was 20.1 milliunits/L; free T 3 and 4 levels were normal. Five days after admission, her laboratory data showed that the serum albumin concentration was 2.3 g/dL and estimated glomerular filtration rate was 83 mL/min/1.73 m^2^. Her frailty exacerbated, and she was completely bedridden. Intervention by the nutritional support team improved her nutritional condition, and her albumin level at discharge was 3.2 g/dL. She was diagnosed with subclinical hypothyroidism, which was treated with levothyroxine sodium hydrate at a dose of 12.5 microgram/day. She could eat nutritionally modified food by herself and was discharged to the previous nursing facility.

### 2.2. White Nails with Sparing of the First Fingernail Bed

**Figure 2:**
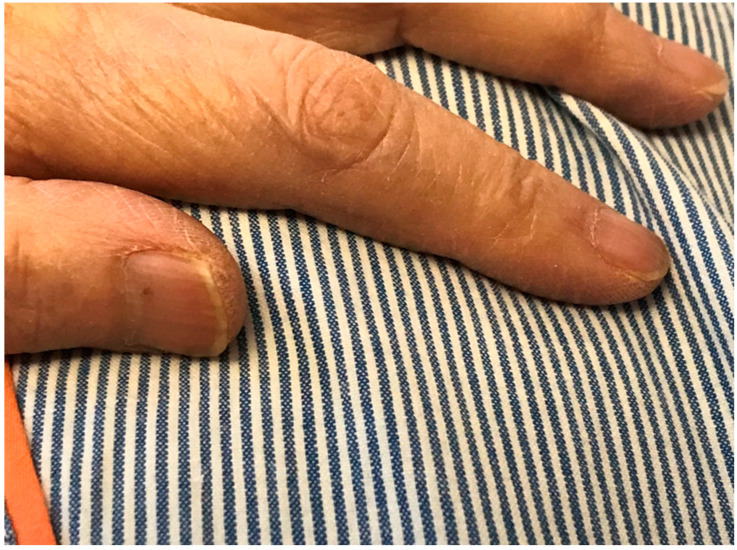
The patient’s white nails with sparing of the first fingernail bed.

An 86-year-old woman came to our hospital complaining of runny nose, muscle pain, mild fever, and appetite loss for one week. Her medical history included aortic stenosis, atrial fibrillation, Alzheimer’s disease, heart failure, and mixed connective tissue diseases requiring prednisolone at a dose of 5 mg; there was no history of liver or renal disease. Her vital signs were normal except for raised temperature (37.2 °C), and she had bilateral proximal muscle pain and tenderness. On admission, her laboratory data showed that the serum albumin concentration was 4.2 g/dL, and the estimated glomerular filtration rate was 34 mL/min/1.73 m^2^. She was diagnosed with viral myositis and treated symptomatically; this resulted in cure, with full recovery of appetite. All fingernail beds of her hands, except for the first fingernail, were white (70%) with brown distal portions (30%); we therefore diagnosed her to have Lindsay’s nail (Figure 2). She did not demonstrate any abnormalities in the levels of minerals and vitamins. However, her blood glucose was 254 mg/dL and hemoglobin A1c was 7.5; this led to a diagnosis of steroid-induced diabetes. One week after admission, her laboratory data showed that the serum albumin concentration was 3.4 g/dL, and the estimated glomerular filtration rate was 64 mL/min/1.73 m^2^. Her clinical condition improved gradually with the addition of metformin at a dose of 500 mg, and she was discharged to her home after 14 days of treatment.

## 3. Discussion

These cases show that serum albumin levels in acute conditions may not be reliable and that white nail findings can show patients’ preclinical status before the acute condition; this includes nutritional conditions. In general, serum albumin levels can indicate patients’ nutritional conditions within several months; this can be used for general nutritional assessment [15]. Nutritional assessment in acute conditions is vital for the evaluation of morbidity and mortality of patients [16]. However, in critical conditions, the systemic volume of the body can be altered, leading to dehydration; this may increase the concentration of the blood, and the values of serum albumin, blood urea nitrogen, and creatinine may be higher than the true values [17]. As demonstrated by this case report, albumin levels at admission can be higher than the usual state after intravascular rehydration. Assessment of the nutritional status of patients in acute care is complicated; on admission, blood data related to nutritional assessment may not show the true picture. Integrating the nail finding for the assessment of nutritional status can be beneficial. Muehrcke’s lines are a well-known finding in the nails of patients with hypoalbuminemia; they are described as transverse white lines on nails. These nail findings can validate the assessment of patients’ nutritional conditions by distinguishing between white nails [18]. Additionally, the differentiation of true and apparent leukonychia is essential, as the findings of Lindsay’s and Terry’s nail are related to changes in the nail bed, and not the nail plate. True leukonychia by infection of fungi should be identified by testing the tissue of nail plates [13].

Nails can be reliable for assessing the preclinical conditions in acute diseases, and the method of assessment using color should be investigated further. Nail beds change gradually, and changes in color occur over several months; these can be referred to as vascular changes in nail beds [19]. Even in acute conditions, nail color can be used to assess preclinical status; this may provide opportunities to investigate unveiled chronic diseases that impair the clinical condition. White discoloration of the fingernails, such as in Terry’s and Lindsay’s nails, can be related to chronic inflammation leading to malnutrition [19,20]. The detection of changes in nail color in the setting of acute conditions can be helpful for predicting patients’ mortality and morbidity. However, the differentiation of Terry’s and Lindsay’s nails may not be evident in older patients, as both findings may be related to aging [19,21,22]. Previous studies are based on younger patients, and the definition of Terry’s and Lindsay’s nails is not clear among older people. Our second patient shows that nail whitening may not appear in all nails and the first fingers of the hands may be spared; this has not been discussed in previous articles [19,21,22]. In addition, the pathophysiology of nail whitening may not be clear. Original articles regarding Terry’s and Lindsay’s nails suggest that nail whitening and the indistinguishable lunula of the nail relate to medical conditions such as malnutrition, congestive heart failure, chronic renal failure, type 2 diabetes mellitus, chronic allograft nephropathy, acute viral hepatitis, vitiligo, and tuberculoid leprosy; however, the fingers that can be involved and the extent of distal discoloration remain unclear [23,24]. Furthermore, older people can have multiple diseases showing findings of white nail. Therefore, while examining nails of older people, the possibilities of multiple diseases need to be considered; this may allow clarification of risk factors of diseases that may modify the clinical course. Therefore, detecting changes in the white nail from the proximal part and the indistinguishable lunula can contribute to effective care of older patients both in hospital and at home. As there is a lack of evidence on nail bed whitening, subsequent studies need to focus on the relationship between clinical outcomes and the presence of white nails.

## 4. Conclusions

White nail can be useful for the evaluation of preclinical conditions in older patients with acute diseases. Further evaluation is needed to establish the relationship between clinical outcomes and the presence of white nail in acute conditions among older patients.

## Data Availability

All relevant data sets in this study are described in the manuscript.
